# Flipped Classroom During Revision Sessions for Second-Year MBBS Students: A Mixed-Methods Study

**DOI:** 10.7759/cureus.112495

**Published:** 2026-07-11

**Authors:** Akhilesh Pathak, Mamta Jadon Pisudde, Pravin M Pisudde, Ravdeep Singh, Anupinder Thind

**Affiliations:** 1 Department of Forensic Medicine, All India Institute of Medical Sciences, Bathinda, IND; 2 Department of Obstetrics and Gynecology, Sardar Patel Institute of Medical Education and Health Sciences, Lucknow, IND; 3 Department of Community and Family Medicine, All India Institute of Medical Sciences, Bathinda, IND; 4 Department of Forensic Medicne, Guru Gobind Singh Medical College and Hospital, Faridkot, IND; 5 Department of Physiology, All India Institute of Medical Sciences, Bathinda, IND

**Keywords:** flipped classroom, revision sessions, second mbbs students, student perception, undergraduate medical teaching

## Abstract

Background: The flipped classroom (FC) approach is increasingly being adopted in medical education; however, students’ responses to its use during revision sessions remain inadequately explored. This study assessed the perceptions, feasibility, and acceptability of the FC approach during revision sessions among second-year Bachelor of Medicine and Bachelor of Surgery (MBBS) students.

Methodology: This mixed-methods, cross-sectional study was conducted among second-year MBBS students at a tertiary care teaching institute. Feedback from the FC-based revision sessions was collected via a structured Google Form with both closed- and open-ended questions. A total of 36 responses were analyzed using frequencies, percentages, the median and interquartile range (IQR), Cronbach's alpha, composite scores, and thematic analysis.

Results: Positive responses were reported for improved engagement and discussion by 22 (61.1%) students, deepened understanding by 21 (58.3%) students, and improved interaction with classmates by 23 (63.9%) students. Feasibility-related responses were also favorable, with 27 (75.0%) students reporting that the instructions were clear, 28 (77.8%) indicating that the pre-class material was effective, 29 (80.6%) considering the outside preparation time manageable, and 35 (97.2%) expressing comfort with the technology used. Preference for the FC over traditional teaching was reported by 14 (38.9%) students. Regarding the overall learning experience, 15 (41.7%) students rated it as fair, while 12 (33.3%) rated it as good. The median scores were 4 (IQR: 3-4) for engagement, 4 (IQR: 2-4) for understanding, 3 (IQR: 2-4) for effective use of class time, and 4 (IQR: 3-4) for interaction. Internal consistency was good, with a Cronbach’s alpha of 0.86 for both the educational value domain and the implementation and acceptability domain.

Conclusions: The FC approach during revision sessions was feasible and positively perceived in terms of engagement, understanding, and peer interaction. However, overall acceptability remained moderate, and preference over traditional teaching methods was limited. Improved session structuring, reduction in the number of presentations, and stronger faculty guidance may enhance the effectiveness of FC-based revision teaching.

## Introduction

The flipped classroom (FC) is a student-centered teaching approach in which initial exposure to learning material occurs before class. At the same time, face-to-face sessions are used for discussion, clarification, and application of knowledge [1]. In medical education, growing interest has been observed in the FC model because it may promote more interactive and accessible learning; however, its effectiveness remains variable across disciplines and study settings [2]. A systematic review in undergraduate health professional education reported that FC may improve academic performance and student satisfaction [3]. Similarly, an earlier systematic review in medical education highlighted inconsistent evidence regarding its effectiveness and emphasized the variable quality of available studies [4].

The FC approach appears particularly relevant in basic medical sciences, as pre-class preparation may allow classroom time to be used for deeper explanation of complex concepts and their clinical application [5]. In India, a randomized crossover study among first-year medical undergraduates found that students preferred the FC format and considered it feasible, although improvements in scores compared with traditional teaching were not clearly superior [6]. Another Indian study in physiology reported that FC was perceived as effective, engaging, and motivating, with improved interaction and active participation among students [7]. More recently, video-based FC teaching in physiology has been evaluated for perception, acceptability, and effectiveness, suggesting that learner response depends not only on the teaching method itself but also on its implementation [8].

However, data regarding the use of FC specifically during revision sessions among second-year Bachelor of Medicine and Bachelor of Surgery (MBBS) students remain limited. Therefore, this study was undertaken to assess the perception, feasibility, and acceptability of FC during revision sessions among second MBBS students.

## Materials and methods

Study design and setting

Second-year MBBS students who attended the FC revision sessions were invited to participate voluntarily in the study. Students were recruited by convenience sampling from those attending the scheduled revision sessions. Those who submitted the feedback questionnaire were included in the study. Students who did not attend the revision sessions, did not submit the feedback questionnaire, or submitted incomplete responses were excluded. A total of 36 complete responses were included in the final analysis.

Participants and selection criteria

Second-year MBBS students who attended the FC revision sessions and submitted the feedback questionnaire were included in the study. Participation was voluntary. Students who did not attend the revision sessions, did not submit the feedback questionnaire, or submitted incomplete responses were excluded. A total of 36 complete responses were included in the final analysis.

FC process

Students were divided into small groups. Each group was assigned a subtopic before the revision session. Relevant learning resources, including recommended study material and references related to the assigned topic, were shared in advance to facilitate self-directed preparation. Students were given adequate time to prepare PowerPoint presentations based on the assigned topic. During the revision session, one representative from each group presented the topic before the class. Faculty members supervised the entire session, facilitated discussion, clarified important concepts, addressed students’ queries, and corrected any misconceptions or knowledge gaps whenever required. Feedback regarding the overall FC experience was collected after completion of the revision sessions using a structured Google Form.

Feedback questionnaire development and validation

Feedback was collected after completion of the FC sessions using a structured Google Form. The questionnaire was specifically developed by the investigators for this study based on a review of the published literature on FC teaching and consensus among the faculty members involved in conducting the revision sessions (Appendix). The questionnaire included both closed-ended and open-ended questions covering engagement, understanding, use of class time, peer interaction, feasibility, acceptability, overall experience, challenges encountered, and suggestions for improvement.

The questionnaire was reviewed independently by three faculty members to establish face validity, content relevance, and clarity of wording. The items were evaluated to ensure that they aligned with the study objectives and were easily understandable by second-year MBBS students. The questionnaire underwent expert review for face and content validation before administration. However, formal quantitative content validation, including calculation of a Content Validity Index (CVI), and pilot testing were not performed. Internal consistency of the questionnaire was assessed after data collection using Cronbach’s alpha.

Questionnaire domains and scoring

The questionnaire consisted of 21 items. Questions 1-4 assessed the educational value of the FC. Questions 5-10 evaluated feasibility, while Questions 11-14 assessed acceptability. Question 15 evaluated the overall learning experience. Questions 16-21 were open-ended questions and were used for qualitative analysis.

Questions 1-4 were rated on a five-point Likert scale, with strongly disagree = 1, disagree = 2, neutral = 3, agree = 4, and strongly agree = 5. Positive responses for these items were defined as agree or strongly agree. Questions 5-14 used a three-point response format (No = 1, Maybe = 2, Yes = 3). Question 15 was scored as Poor = 1, Fair = 2, Good = 3, and Excellent = 4. Higher scores represented more favorable perceptions of the FC approach.

Composite scores were calculated by summing the responses within each domain. The educational value score was calculated using Questions 1-4, the feasibility score using Questions 5-10, and the acceptability score using Questions 11-14. Higher composite scores indicated more positive perceptions regarding the educational effectiveness, feasibility, and acceptability of the FC approach.

Qualitative analysis

Responses to the open-ended questions (Questions 16-21) were analyzed using thematic analysis. Two investigators independently read all responses multiple times to familiarize themselves with the data and generated initial codes. Similar codes were grouped into broader categories to identify recurring themes. Any differences in coding or theme allocation were resolved through discussion until consensus was achieved. The final themes were reviewed against the original responses and the study objectives to ensure consistency and accuracy before being reported with representative interpretations.

Statistical analysis

Data were analyzed using RStudio (Posit PBC, Boston, MA). Categorical variables were summarized as frequencies and percentages, whereas ordinal variables were expressed as median and interquartile range (IQR). Selected key proportions were reported with 95% Wilson confidence intervals. Composite scores for educational value, feasibility, and acceptability were calculated as described above. Internal consistency of the educational value domain and the implementation and acceptability domain was assessed using Cronbach’s alpha, with higher values indicating better internal consistency of the questionnaire. As this was an exploratory educational evaluation involving all eligible students who voluntarily completed the questionnaire, no formal sample size calculation was performed.

## Results

Overall, participants showed a positive perception of the FC approach. Improved interaction with classmates received the highest agreement, with 19 (52.8%) participants agreeing and 4 (11.1%) strongly agreeing. This was followed by improved engagement and discussion, where 17 (47.2%) agreed, and 5 (13.9%) strongly agreed. Similarly, 16 (44.4%) agreed, and 5 (13.9%) strongly agreed that FC deepened understanding of the topic. Perception of better use of class time was comparatively lower, with 12 (33.3%) agreeing and 2 (5.6%) strongly agreeing, while 10 (27.8%) remained neutral. Overall, responses indicate generally favorable perceptions, with the strongest agreement for peer interaction and engagement (Table 1).

**Table 1:** Responses to 5-point Likert scale items on the flipped classroom during revision sessions among second-year MBBS students (N = 36).

Item	Strongly disagree, *n* (%)	Disagree, *n* (%)	Neutral, *n* (%)	Agree, *n* (%)	Strongly agree, *n* (%)
Improved engagement and discussion	3 (8.3)	5 (13.9)	6 (16.7)	17 (47.2)	5 (13.9)
Deepened understanding of the topic	1 (2.8)	11 (30.6)	3 (8.3)	16 (44.4)	5 (13.9)
Better use of class time	2 (5.6)	10 (27.8)	10 (27.8)	12 (33.3)	2 (5.6)
Improved interaction with classmates	2 (5.6)	3 (8.3)	8 (22.2)	19 (52.8)	4 (11.1)

Overall, participants demonstrated high acceptability of the FC approach. Thirty-five (97.2%) participants reported comfort with technology, while manageable outside preparation time was reported by 29 (80.6%). The pre-class material was reported to be effective by 28 (77.8%) students, and the instructions were reported to be clear by 27 (75.0%) students. Group work was engaging for 18 (50.0%) participants. However, preference for FC over traditional teaching methods was lower, with 14 (38.9%) participants responding “Yes.” A total of 19 (52.8%) participants supported future participation, while 16 (44.4%) supported future implementation, and 15 (41.7%) felt it suited their learning style. Overall, participants demonstrated strong acceptability, with moderate variability in preference for adoption over traditional teaching (Table 2).

**Table 2:** Responses to “yes/maybe/no” items on flipped classroom during revision sessions among second-year MBBS students (N = 36).

Item	No, *n* (%)	Maybe, *n* (%)	Yes, *n* (%)
Engaging group work	8 (22.2)	10 (27.8)	18 (50.0)
Clear instructions	6 (16.7)	3 (8.3)	27 (75.0)
Effective pre-class material	4 (11.1)	4 (11.1)	28 (77.8)
Manageable outside preparation time	3 (8.3)	4 (11.1)	29 (80.6)
Balanced workload	8 (22.2)	6 (16.7)	22 (61.1)
Comfort with technology	0 (0.0)	1 (2.8)	35 (97.2)
Preference for FC over traditional teaching	16 (44.4)	6 (16.7)	14 (38.9)
Future participation	10 (27.8)	7 (19.4)	19 (52.8)
Suitability to learning style	14 (38.9)	7 (19.4)	15 (41.7)
Future implementation of FC	13 (36.1)	7 (19.4)	16 (44.4)

Overall, most participants reported a positive perception of the FC approach. The majority rated it as fair (15, 41.7%), followed by good (12; 33.3%). A smaller proportion rated it as excellent (4, 11.1%), while 5 (13.9%) reported a poor rating. Overall, findings indicate a generally favorable perception of the intervention (Table 3).

**Table 3:** Overall student perceptions of the flipped classroom during revision sessions (N = 36).

Response category	*n* (%)
Poor	5 (13.9)
Fair	15 (41.7)
Good	12 (33.3)
Excellent	4 (11.1)

Overall, participants reported generally positive perceptions of engagement-related aspects. Improved engagement and discussion had a median score of 4 (IQR 3-4), and improved interaction with classmates also had a median of 4 (IQR 3-4), indicating consistently high ratings. Deepened understanding showed favorable responses with a median of 4 (IQR 2-4), while better use of class time received a moderate rating of 3 (IQR 2-4). However, overall experience was relatively less favorable, with a median score of 2 (IQR 2-3), indicating mixed satisfaction despite positive ratings in specific domains (Table 4).

**Table 4:** Median and interquartile ranges of ordinal items on the flipped classroom during revision sessions among second-year MBBS students (N = 36). Scores for Q1-Q4 were coded from 1 to 5, with Strongly disagree = 1, Disagree = 2, Neutral = 3, Agree = 4, and Strongly agree = 5. Overall experience was coded from 1 to 4, with Poor = 1, Fair = 2, Good = 3, and Excellent = 4. A higher score indicates a more favorable response. IQR, interquartile range

Item	Median	IQR
Improved engagement and discussion	4	3-4
Deepened understanding of the topic	4	2-4
Better use of class time	3	2-4
Improved interaction with classmates	4	3-4
Overall experience	2	2-3

The questionnaire demonstrated good internal consistency, with a Cronbach’s alpha of 0.86 (95% CI: 0.76-0.92) for the educational value domain and 0.86 (95% CI: 0.77-0.92) for the implementation and acceptability block. The educational value domain showed a median composite score of 14.5 (IQR 11.0-16.0), indicating generally favorable perceptions. Feasibility demonstrated a higher median score of 16.0 (IQR 14.0-18.0; range 8-18), suggesting strong perceived practicality. Acceptability showed a comparatively lower median score of 8.0 (IQR 5.5-12.0), indicating more variable responses in this domain. Overall, the results suggest good reliability with generally positive perceptions of feasibility and educational value (Table 5).

**Table 5:** Internal consistency of questionnaire domains and composite domain scores for the flipped classroom during revision sessions among second-year MBBS students (N = 36). The educational value score was calculated by summing Q1-Q4. The feasibility score was calculated by summing Q5-Q10. The acceptability score was calculated by summing Q11-Q14. Q1-Q4 were scored from 1 to 5. Q5-Q14 were scored as No = 1, Maybe = 2, and Yes = 3. A higher composite score indicated a more favorable response. Cronbach's alpha was calculated for the educational value block and the implementation and acceptability block. IQR, interquartile range

Domain	Items	Cronbach’s alpha	Median composite score	IQR	Observed range
Educational value	Q1–Q4	0.86	14.5	11.0-16.0	5-20
Feasibility	Q5-Q10	-	16.0	14.0-18.0	8-18
Acceptability	Q11-Q14	-	8.0	5.5-12.0	4-12
Implementation and acceptability block	Q5-Q14	0.86	-	-	-

Overall, positive responses were highest for technology-related acceptability, with 35/36 (97.2%, 95% CI: 85.8-99.5) participants reporting comfort with technology. Among pedagogical outcomes, improved interaction with classmates showed the highest agreement at 23/36 (63.9%, 95% CI: 47.6-77.5), followed by improved engagement and discussion at 22/36 (61.1%, 95% CI: 44.9-75.2), and deepened understanding of the topic at 21/36 (58.3%, 95% CI: 42.2-72.9). In contrast, perceptions were lower for better use of class time and preference over traditional teaching, both reported by 14/36 (38.9%, 95% CI: 24.8-55.1). Overall, findings indicate strong acceptability with moderate variability in perceived educational advantage (Table 6). 

**Table 6:** Selected key proportions with 95% Wilson confidence intervals for the flipped classroom during revision sessions among second-year MBBS students (N = 36). CI, confidence interval

Variable	*n*/*N*	Proportion (%)	95% CI
Positive response for improved engagement and discussion	22/36	61.1	44.9-75.2
Positive response for deepened understanding of the topic	21/36	58.3	42.2-72.9
Positive response for improved interaction with classmates	23/36	63.9	47.6-77.5
Positive response for better use of class time	14/36	38.9	24.8-55.1
Preferred the flipped classroom over traditional teaching	14/36	38.9	24.8-55.1
Comfortable with technology	35/36	97.2	85.8-99.5

Selected key proportions with 95% Wilson confidence intervals are shown in Table 6. Positive response for improved engagement and discussion was 61.1% (95% CI: 44.9-75.2), for deepened understanding was 58.3% (95% CI: 42.2-72.9), and for improved interaction with classmates was 63.9% (95% CI: 47.6-77.5). Positive response for better use of class time and preference over traditional teaching was 38.9% each. Comfort with technology was 97.2% (95% CI: 85.8-99.5). 

Thematic analysis of open-ended responses identified six main themes: active learning and engagement, peer interaction, better understanding, time and preparation burden, uneven participation, and need for stronger faculty guidance. Students reported that flipped revision promoted active preparation, group discussion, and peer interaction. However, some students felt that the method required more preparation time and that multiple presentations made the session lengthy. Uneven participation was also noted, with some responses suggesting that presenters benefited more than non-presenting students. Students suggested fewer presentations, better time control, and more faculty-led discussions after each presentation. Technical issues were not a major concern. Thematic analysis of open-ended feedback is shown in Table 7. The main favorable themes were active learning, peer interaction, and better understanding. Students felt that the flipped revision method encouraged preparation before class and improved discussion during the session. Group work also helped students interact with classmates during preparation and in-class activity. The main concerns were time burden, preparation load, and uneven participation. Some students felt that multiple presentations made the session lengthy. A few responses suggested that presenters benefited more than non-presenting students. Students also felt that faculty guidance was needed after presentations to clarify difficult points and correct gaps in understanding. Technical difficulty was not a major theme.

**Table 7:** Themes from open-ended feedback on the flipped classroom during revision sessions among second-year MBBS students (N = 36).

Theme	Main finding	Representative interpretation
Active learning and engagement	Students felt that the method promoted active participation and discussion	Flipped revision encouraged students to prepare and participate rather than only listen passively.
Peer interaction	Group work improved interaction with classmates	Students perceived better peer discussion during preparation and in-class activity.
Better understanding	Some students felt that topic preparation and presentation improved their understanding.	The method helped students revise the topic actively and clarify selected areas.
Time and preparation burden	Preparation outside class and multiple presentations were perceived as time-consuming.	The format required extra time and was not accepted equally by all students.
Uneven participation	Some students felt that presenters benefited more than non-presenting students.	Audience participation was not uniform, and some classmates remained passive.
Need for faculty guidance	Students suggested more faculty moderation and a summary after presentations.	Faculty input was considered important for correcting gaps and clarifying difficult concepts.
Technical issues	Technical difficulty was not a major theme.	Most students were comfortable using technology for flipped classroom activities

## Discussion

In this study, the FC approach during revision sessions was perceived favorably in terms of engagement, understanding, and peer interaction, although overall acceptability remained moderate. Positive responses were reported for engagement and discussion (22, 61.1%), improved understanding (21, 58.3%), and interaction with classmates (23, 63.9%). In contrast, preference for FC over traditional teaching methods was lower (14, 38.9%), suggesting that while students appreciated the active learning components, they were less convinced about replacing conventional teacher-led revision methods. Overall, these findings suggest that FC during revision sessions was feasible and educationally beneficial for promoting participation and interaction; however, concerns regarding acceptability and implementation persisted.

In this study, FC during revision sessions was perceived favorably in terms of engagement, understanding, and peer interaction, although overall acceptability remained moderate. Positive responses were reported for engagement and discussion (22, 61.1%), improved understanding (21, 58.3%), and interaction with classmates (23, 63.9%). In contrast, preference for FC over traditional teaching methods was lower (14, 38.9%), and the overall experience was mainly rated as fair to good. These findings suggest that students appreciated the active learning components of the sessions but were less convinced about fully replacing conventional teacher-led revision methods. Similar findings were reported by Mengesha et al. [9], where 80% of undergraduate medical students reported improved engagement, 76% reported better understanding, 66% preferred the flipped method, and overall satisfaction reached 76%. Khadawardi et al. [10] also demonstrated improvement following the flipped intervention, with mean test scores increasing from 13.25 ± 2.36 to 16.08 ± 1.50 and impact scale scores rising from 41.91 ± 4.90 to 69.71 ± 9.72.

The quantitative findings of the present study are further supported by previous reports showing that well-structured FC formats can enhance comprehension and active participation. Agarwal and Joshi [11] reported high student ratings for handout quality (4.57 ± 0.42), comprehension and retention (4.28 ± 0.44), and satisfaction and engagement (4.49 ± 0.41). In the same study, 96% of students stated that scenario-based discussions improved understanding, while 91% reported that multiple-choice questions (MCQs) reinforced key concepts. Paralikar et al. [12] similarly demonstrated higher post-test performance with FC compared with traditional teaching in physiology, with scores of 5.36 ± 1.69 versus 4.94 ± 1.34. Consistent with these findings, median scores in the present study were favorable for engagement, understanding, and interaction. Collectively, these findings support the role of FC in promoting active learning, although perceived benefit may depend substantially on session organization and implementation.

An important finding of the present study was that effective utilization of class time was not strongly perceived by students. Qualitative responses repeatedly highlighted concerns regarding preparation burden, excessive presentations, and time constraints. These findings are important because FC may not always be perceived as efficient, particularly when both pre-class preparation and in-class activities are extensive. Li et al. [13] demonstrated that a partially flipped physiology classroom promoted a deeper learning approach, whereas traditional teaching was more strongly associated with surface learning. This suggests that FC may improve the quality of learning rather than simply making sessions easier for students. Ji et al. [14] further reported that although FC increased time investment in physiology learning, it did not adversely affect performance in concurrent subjects and was associated with improved subsequent performance in pathophysiology, pathology, pharmacology, diagnostics, and internal medicine. Together, these studies support the educational value of FC. However, the present findings indicate that students may perceive revision-session formats less favorably when associated with increased workload and time pressure.

Qualitative findings from the present study also suggested that presenters benefited more than non-presenting classmates, while faculty moderation was perceived as insufficient. This may explain why feasibility scores were higher than acceptability scores. Alzaabi et al. [15] reported that 20 of 25 students considered peer learning valuable; however, supervised teaching resulted in significantly better performance than peer learning alone, with mean scores of 17.33 ± 2.57 versus 14.20 ± 3.25 (*P* = 0.003). The authors also concluded that faculty-supported teaching sessions should precede peer-learning activities. Similarly, Chan et al. [16] evaluated 205 medical students, of whom 131 completed the questionnaire. For bag-mask ventilation training, students who watched demonstration videos before class achieved significantly higher scores than those who did not (7.8 ± 1.0 vs. 6.3 ± 1.7, *P* < 0.01). In intravenous cannulation training, students who viewed instructional videos beforehand required fewer peer interventions during practice (2.9 ± 1.8 vs. 4.3 ± 2.9, *P* < 0.05). Together with the present findings, these studies suggest that pre-class preparation is beneficial, but peer-led learning is more effective when accompanied by adequate faculty guidance.

Additional evidence from more structured clinical and pharmacology-based FC models further supports this interpretation. Liebert et al. [17] evaluated a simulation-based FC curriculum in a surgery clerkship. They reported very high acceptance, with 90% of students rating the curriculum as excellent or outstanding, 95% recommending its continuation, and 84% supporting its application in other clerkships. Angadi et al. [18] also demonstrated significant academic benefit in pharmacology, where end-of-module scores were higher in the FC group than in the conventional small-group teaching group (15.53 ± 3.76 vs. 9.61 ± 3.90). Furthermore, 96% of students reported that interactive in-class activities enhanced learning, and all participants expressed interest in additional FC sessions in the future. Compared with these studies, the overall experience in the present study was less favorable. This difference likely reflects limitations in session design rather than limitations of the FC concept itself. In revision settings, reducing the number of presentations, using smaller groups, increasing faculty moderation, and incorporating MCQs, quizzes, or guided discussions may substantially improve acceptability.

Limitations and strengths

This was a single-center study with only 36 students, so the findings have limited generalizability. The study assessed student perception after revision sessions and did not compare academic performance before and after the intervention. There was no control group using traditional revision teaching. Faculty members reviewed the feedback questionnaire, but no separate pilot testing or formal pre-study psychometric validation was done. The qualitative findings were based on short open-ended responses, so detailed student views may not have been fully captured. In addition, formal quantitative questionnaire validation, including calculation of a CVI, was not performed, and participant recruitment was based on voluntary participation rather than probability sampling. Despite these limitations, the study provides useful feedback on the feasibility and acceptability of the FC during revision teaching. 

This study provides context-specific evidence regarding the use of FC during revision sessions among second-year MBBS students. Although FC demonstrated good feasibility and educational value, overall acceptability remained moderate. The mixed-methods design helped clarify this difference. Quantitative findings showed favorable engagement and interaction, whereas qualitative responses identified preparation burden, passive audience participation, unequal involvement, and insufficient faculty supervision as key barriers. Overall, the findings suggest that FC in revision teaching may be more effective when implemented as a moderated small-group discussion strategy rather than as a rapid sequence of student presentations.

## Conclusions

The FC approach during revision sessions was feasible in this group of second-year MBBS students. It was associated with favorable perceptions regarding engagement, understanding, and peer interaction. However, acceptability remained moderate, and preference over traditional teaching methods was limited. Improved planning, a reduced number of presentations, and stronger faculty guidance may enhance its effectiveness in routine revision teaching.

## Appendices

Appendix: Questionnaire

**Table 8:** Feedback questionnaire used for flipped classroom revision sessions

Question number	Questionnaire item	Response option	Domain / use
Q1	Flip class method is useful for engaging the students and good in-class activity and discussions.	5-point Likert scale	Educational value
Q2	Did you feel that the flip class method is effective to deepen your understanding of the topics?	5-point Likert scale	Educational value
Q3	The flip class method is better to use your in-class time effectively.	5-point Likert scale	Educational value
Q4	Flipped classroom model was good and increased your interaction with classmates during in-class activities.	5-point Likert scale	Educational value
Q5	Did you feel flip class is more engaging in group work compared to traditional classroom settings?	Yes / Maybe / No	Feasibility
Q6	The instructions and materials provided by the teacher before flip class were clear and easy to understand.	Yes / Maybe / No	Feasibility
Q7	The pre-class materials, videos or readings in preparing you for in-class activities were effective.	Yes / Maybe / No	Feasibility
Q8	You were able to manage your time outside class to complete pre-class assignments.	Yes / Maybe / No	Feasibility
Q9	Did you find it easy to balance the workload from other courses with the flipped classroom approach?	Yes / Maybe / No	Feasibility
Q10	Were you comfortable with using technology for the flipped classroom activities?	Yes / Maybe / No	Feasibility
Q11	Do you prefer the flipped classroom model over traditional teaching methods?	Yes / Maybe / No	Acceptability
Q12	Would you like to participate in flip class in future also?	Yes / Maybe / No	Acceptability
Q13	Did the flipped classroom model suit your learning style?	Yes / Maybe / No	Acceptability
Q14	Do you think that the flip class method should be implemented for teaching in future?	Yes / Maybe / No	Acceptability
Q15	How would you describe your experience with flipped learning compared to traditional teaching methods?	Poor / Fair / Good / Excellent	Overall experience
Q16	Were there any challenges you encountered with the flipped classroom model?	Open-ended	Qualitative feedback
Q17	If so, what specific aspects of the flipped model were difficult for you?	Open-ended	Qualitative feedback
Q18	What are your overall thoughts on the flipped classroom approach?	Open-ended	Qualitative feedback
Q19	What suggestions do you have to improve the effectiveness of the flipped classroom approach?	Open-ended	Qualitative feedback
Q20	Were there any technical issues that hindered your learning experience?	Open-ended	Qualitative feedback
Q21	Are there specific changes or adjustments you would recommend?	Open-ended	Qualitative feedback

## References

[1] Liu J, Wu Z, Lan Y-Z, Chen W-J, Wu B-X, Chen W-T, Wu H-T (2024). Flipped classroom in physiology education: where are we and where are we heading?. Front Educ.

[2] Spaic D, Bukumiric Z, Rajovic N (2025). The flipped classroom in medical education: systematic review and meta-analysis. J Med Internet Res.

[3] Naing C, Whittaker MA, Aung HH, Chellappan DK, Riegelman A (2023). The effects of flipped classrooms to improve learning outcomes in undergraduate health professional education: a systematic review. Campbell Syst Rev.

[4] Chen F, Lui AM, Martinelli SM (2017). A systematic review of the effectiveness of flipped classrooms in medical education. Med Educ.

[5] Patkar KU, Patkar US, Kolte VS (2021). Efficacy of flipped classroom method in teaching-learning physiology. Indian J Physiol Pharmacol.

[6] Prabhavathi K, KalyaniPraba P, Rohini P, Selvi KT, Saravanan A (2024). Flipped classroom as an effective educational tool in teaching physiology for first-year undergraduate medical students. J Educ Health Promot.

[7] Malhotra AS, Bhagat A (2023). Flipped classroom for undergraduate medical students in India: are we ready for it?. Adv Physiol Educ.

[8] Kottath Veetil P, Kollukkad Mani M, Arja SB, Paramban S, Kattambally PA, Fatteh R, Arja SB (2025). Student perceptions and effectiveness of video-based flipped classroom for improving medical physiology teaching at AUSOM. Adv Med Educ Pract.

[9] Mengesha AK, Ayele HS, Misker MF, Beyna AT (2024). Assessing the effectiveness of flipped classroom teaching-learning method among undergraduate medical students at Gondar University, College of Medicine and Health Sciences: an interventional study. BMC Med Educ.

[10] Khadawardi K, Mirdad D, Nasief H (2025). Evaluating medical student engagement in flipped classrooms: insights on motivation and peer learning. J Med Educ Curric Dev.

[11] Agarwal M, Joshi P (2025). Effectiveness of modified flipped classrooms integrating scenario-based questions, multiple-choice question assessments, and mind maps in blood physiology. Cureus.

[12] Paralikar S, Shah CJ, Joshi A, Kathrotia R (2022). Acquisition of higher-order cognitive skills (HOCS) using the flipped classroom model: a quasi-experimental study. Cureus.

[13] Li D, Zhang C, Fang J (2025). Exploring physiological knowledge through models: a pathway to innovation in medical education. Preprint.

[14] Ji M, Luo Z, Feng D, Xiang Y, Xu J (2022). Short- and long-term influences of flipped classroom teaching in physiology course on medical students’ learning effectiveness. Front Public Health.

[15] Alzaabi S, Nasaif M, Khamis AH, Otaki F, Zary N, Mascarenhas S (2021). Medical students’ perception and perceived value of peer learning in undergraduate clinical skill development and assessment: mixed methods study. JMIR Med Educ.

[16] Chan E, Botelho MG, Wong GT (2021). A flipped classroom, same-level peer-assisted learning approach to clinical skill teaching for medical students. PLoS One.

[17] Liebert CA, Mazer L, Bereknyei Merrell S, Lin DT, Lau JN (2016). Student perceptions of a simulation-based flipped classroom for the surgery clerkship: a mixed-methods study. Surgery.

[18] Angadi NB, Kavi A, Shetty K, Hashilkar NK (2019). Effectiveness of flipped classroom as a teaching-learning method among undergraduate medical students - an interventional study. J Educ Health Promot.

